# EVIDENCE THAT TOMORROW’S OLDER POPULATION WILL BE LESS FIT THAN TODAY: A NATIONAL STUDY, 2003–2022

**DOI:** 10.1093/geroni/igae098.2874

**Published:** 2024-12-31

**Authors:** David Rehkopf, Frank Furstenberg, Christian Jackson, John Rowe

**Affiliations:** Stanford University, Palo Alto, California, United States; University of Pennsylvania, Philadelphia, Pennsylvania, United States; Stanford University, Stanford, California, United States; Columbia University, New York, New York, United States

## Abstract

Entering retirement age with a high level of health, well-being, and functioning is critical for remaining healthy and engaged after retirement and into later life. While recent stagnating trends for mortality have been well documented in pre-retirement age groups, there has been less attention to trends in morbidity, and in particular, what they are like across different levels of income. We use data from the Centers for Disease Control and Prevention Behavioral Risk Factor Surveillance System that includes self-reported data on wellbeing and functioning on more than seven million individuals from 2003 to 2022, to examine how physical health, mental health and functional health have changed over time by age group and income level. We find there are dramatic differences (up to 10 more days of morbidity in the last 30 days) by level of income, and that over this period of time there are increases in the number of days with bad health and functioning for the poorest and lower-middle income groups. These declines are most notable in ages that are pre-retirement years, age 50 to 59. None of these trends or levels were explained by race, BMI, or smoking, although we show substantial differences in these trends across U.S. States. If current national trends persist, the current age 50 to 59 year old population will be entering retirement ages with substantially worse health than previous generations, which has major implications for labor force participation, Medicare expenses and Social Security.

